# A Rare Case of Antenatal Bilateral Multicystic Dysplastic Kidney Disease: An Unusual Presentation in a Neonate

**DOI:** 10.1002/ccr3.70407

**Published:** 2025-04-07

**Authors:** Samina Chaki, Ronald Mclarty, Isack Mzolo, Shaffin Rajan, Hilary Chipongo

**Affiliations:** ^1^ Department of Pediatrics and Child Health Shree Hindu Mandal Hospital Dar es Salaam Tanzania; ^2^ Department of Radiology and Diagnostic Imaging Shree Hindu Mandal Hospital Dar es Salaam Tanzania; ^3^ Critical Care Department Shree Hindu Mandal Hospital Dar es Salaam Tanzania

**Keywords:** congenital anomaly, multicystic dysplastic kidney, sub‐Saharan Africa, ultrasonography

## Abstract

This report highlights a deadly presentation of Multicystic dysplastic kidney (MCDK) diagnosed in utero and confirmed after 6 weeks post‐delivery by ultrasonography. In this case, we present a neonate followed for a period of 6 weeks after delivery without any notable complications. From the literature point of view, this condition is incompatible with life and there are few cases reported in sub‐Saharan Africa. There is no definitive treatment for such a case; only watchful waiting and serial renal function tests are used to monitor complications to date. Hence, this case opens a window for other researchers to evaluate this course, especially in resource‐limited settings.

**Summary**

- Multicystic dysplastic kidney (MCDK) disease is lethal, as reported in the literature; few cases are reported, and prognostic factors are still not well understood.
- This case highlights a severe form of MCDK reported in Africa.

**Abbreviations**
MCDK: multicystic dysplastic kidney
PMTCT‐2: prevention of mother‐to‐child transmission
UTI: urinary tract infection
VUR: vesicoureteral reflux

## 1 Introduction

Multicystic dysplastic kidney (MCDK) disease is a congenital kidney and urinary tract anomaly seen in 1 out of 4300 live births, commonly affecting the left side and with a male predominance [1]. The condition is generally unilateral and is characterized by multiple non‐communicating cysts in the renal parenchyma [1]. The condition is more commonly seen in Caucasians and is accompanied by genitourinary anomalies like vesical‐ureteral reflux [2]. In sub‐Saharan Africa, there are no cases reported to the best of our knowledge involving antenatal multicystic kidney disease. Also, current literature shows bilateral involvement as a rare entity and usually incompatible with life [2]. Herein we present a case of bilateral MCDK diagnosed in utero and delivered uneventfully with normal renal function tests.

## 2 Case Presentation

A healthy one‐day‐old male patient was noticed to have an abnormal urethral opening by his mother soon after delivery. He was delivered by caesarean section due to poor progress of labour, weighing 3.5 kg and scoring 9 and 10 in the first and fifth minutes, respectively. The mother was prime gravida, PMTCT‐2, attended 6 antenatal clinic visits, and was fully immunized, receiving all supplements in the course of pregnancy. Other antenatal history and family social history were unremarkable. Prior to delivery, the patient was diagnosed with MCDK and confirmed after birth.

## 3 Investigations

**FIGURE 1:**
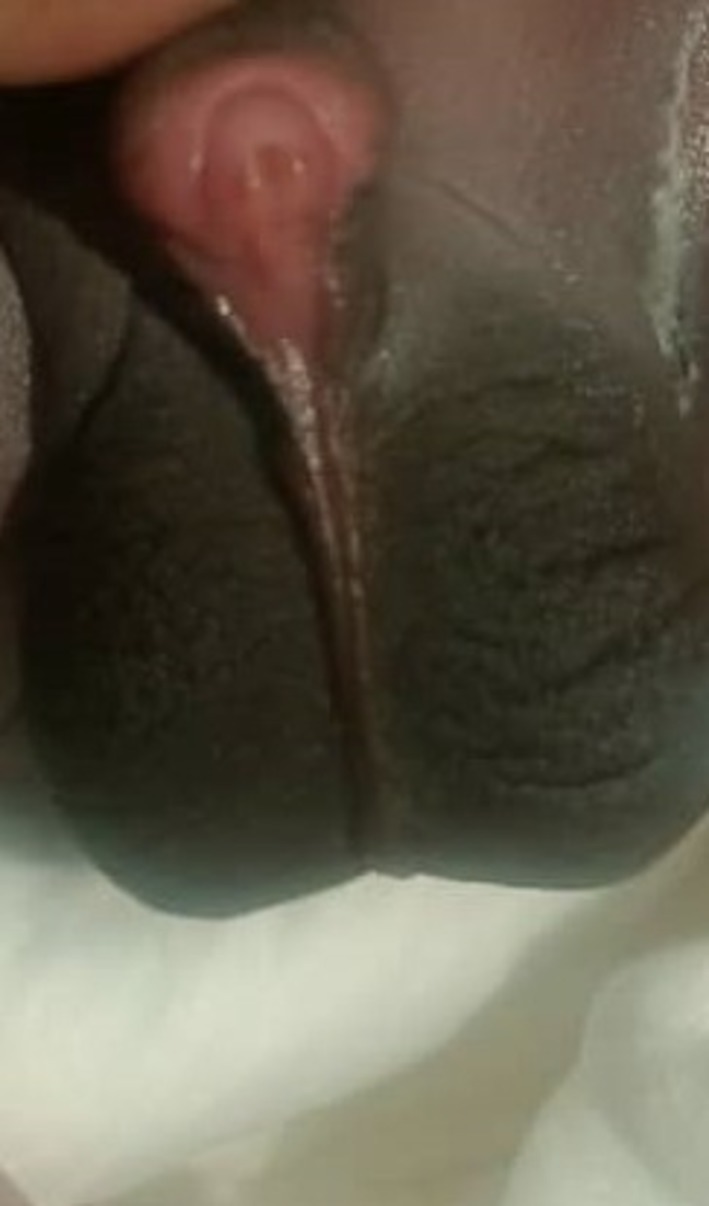
Image showing dorsal hypospadias on a day‐one neonate.

**FIGURE 2:**
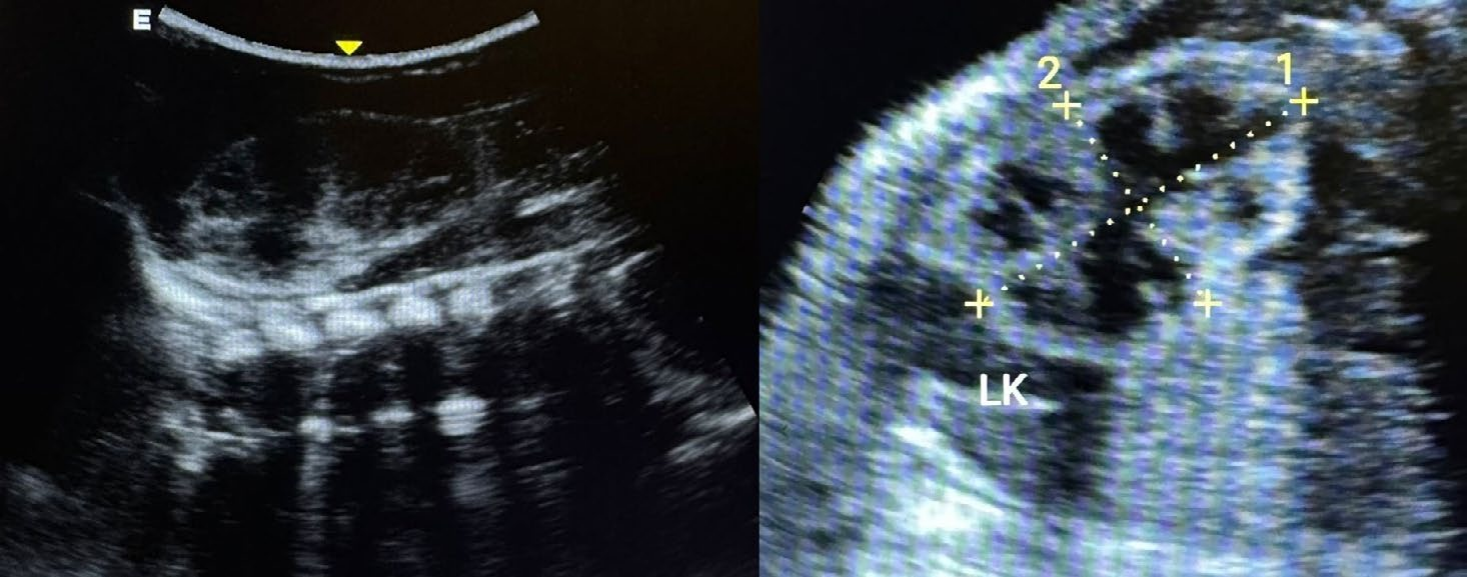
In utero ultrasonography showing bilateral MCDK.

**FIGURE 3:**
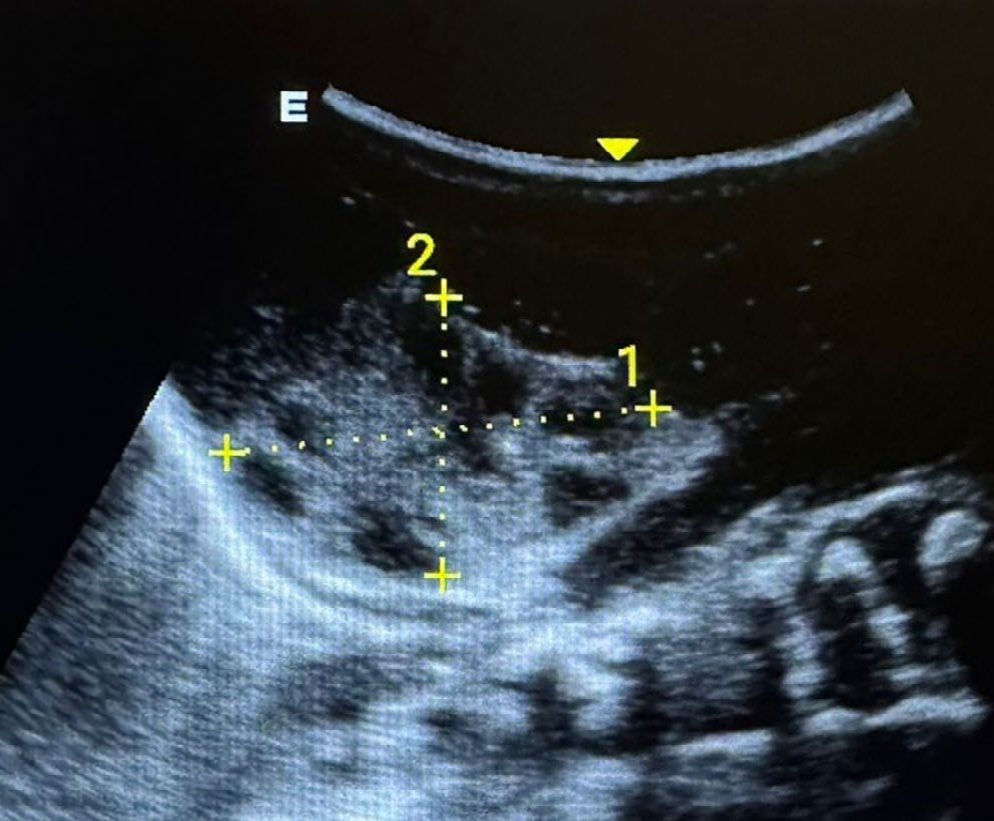
Ultrasonography images 1 day after delivery showing multiple cysts in the kidney.

**FIGURE 4:**
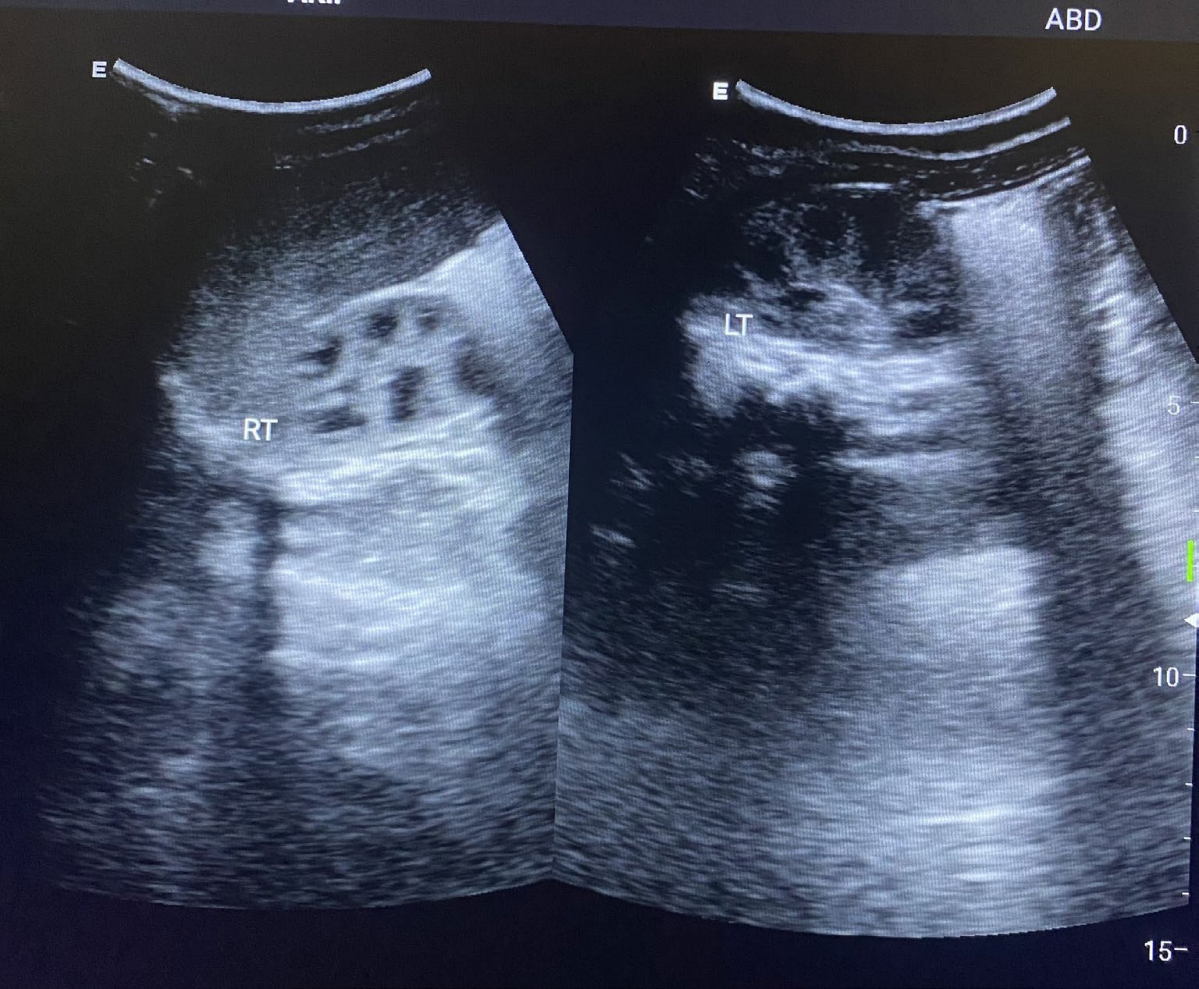
Ultrasonography after 6 weeks post‐delivery showing evidence of multiple cysts in the kidneys.

The patient had normal renal function tests for his age. On examination, hypospadias was confirmed as seen in Figure 1. In utero ultrasonography showing bilateral MCDK is shown in Figure 2. Soon after delivery, another ultrasound was done, which is seen in Figure 3. The patient was followed on a monthly basis, and after 6 weeks, repeat renal function tests were done, which were unremarkable. Figure 4 shows an ultrasound scan after 6 weeks post‐delivery.

## 4 Discussion

MCDK presents with unilateral involvement, affecting the left‐sided kidney, and is usually asymptomatic [2]. In utero diagnosis has aided in the confirmation of the pathology even before the newborn is delivered. A review of the literature has shown that bilateral involvement of MCDK is lethal and has been associated with life incompatibility [2]. Studies have shown several complications of MCDK which commonly are UTI (urinary tract infections), hypertension, and renal malignancy [3]. Very few cases are associated with abdominal or flank pain and respiratory distress due to the pressure effect of the abnormal kidney [3]. A review done by Kara et al. in 2018 involving 128 patients showed the prevalence of UTI to be 3.9% which was not higher than the overall incidence of pediatric UTI [4]. This case highlights a unique presentation of MCDK reported in sub‐Saharan Africa, no similar cases reported previously in the same geographical location.

The diagnosis of MCDK is made before birth as early as the 15th week of gestation when many tiny cysts begin to appear [5]. This patient was attending clinics at other health centers and the initial diagnosis was missed the pathology was seen on ultrasonography during the late 30's week of gestation where bilateral hydronephrosis was incidentally found on the fetus scan. Senior review and repeated scan at 39th‐week gestation revealed the diagnosis of bilateral MCDK. The child was delivered uneventfully, an abdominal ultrasound was done post‐delivery, and confirmed again the diagnosis of MCDK with normal renal and liver function tests. Currently, the child is followed up closely at least once every 3 months monitoring renal function tests and milestones.

To date, conservative management with follow‐up is the mainstay of treatment [6]. Serial ultrasonography images are to be taken during regular clinic follow‐up until resolution has occurred [6]. Nephrectomy is indicated only if the enlarged kidney fails to regress, hence causing obstructive uropathy, the development of malignancy, or MCDK becomes the cause of hypertension or urosepsis [2].

The literature review has shown that MCDK is associated with various genitourinary anomalies such as VUR (vesicoureteral reflux), pelviureteric stenosis, ureterocele, and cryptorchidism [3]. VUR has been seen to be the most common congenital anomaly accounting for more than 20% on the contralateral side of the affected kidney in several literature reviews [7, 8]. A review in Europe in 2022 by Kopac showed that cryptorchidism is the most associated anomaly accounting for 24% of all anomalies, the second being VUR, which was 16.3% (associated anomalies and complications of MCDK) [3]. This opens room for more review from different parts of the globe as the entity is still not commonly reported. Although not common, a similar presentation with hypospadias was reported in Turkey in 2018 following a review of 128 patients, in which one of the patients presented with hypospadias as seen in our patient.

The prognosis of MCDK is reported to be favorable as there is involution of the cysts after delivery of the child [9]. This is concerning unilateral involvement, which is the common presentation of this congenital anomaly. A review done in Asia by Alamir et al. in 2023, in which a retrospective study for 6 years involving 57 patients with MCDK was reviewed, found that out of these, 7 had bilateral MCDK in utero and were excluded from the study and all of them died antenatally and a few weeks post‐delivery due to hypoplastic lungs [9]. This report highlights the prognostic consideration of bilateral MCDK for a patient who had thrived 6 weeks post‐delivery.

## 5 Conclusion

In sub–Saharan Africa there are no cases reported involving MCDK to date. Hence the exact prevalence of the condition is still not certain. This article highlights the importance of regular antenatal diagnosis of this condition and its associated morbidity. Clinicians are cautioned to be vigilant to screen for such pathologies like this.

## Author Contributions

**Samina Chaki:** writing – original draft. **Ronald Mclarty:** writing – original draft. **Isack Mzolo:** data curation. **Shaffin Rajan:** writing – review and editing. **Hilary Chipongo:** supervision.

## Ethics Statement

The case report was conducted following ethical standards, and patient confidentiality was maintained.

## Consent

Written consent for publication was obtained, and if needed, may be provided to the editor‐in‐chief.

## Conflicts of Interest

The authors declare no conflicts of interest.

## Data Availability

The data used to support the findings of this study are available from the corresponding author upon reasonable request.
